# Epigenetic Inactivation of the *miR-124-1* in Haematological Malignancies

**DOI:** 10.1371/journal.pone.0019027

**Published:** 2011-04-22

**Authors:** Kwan Yeung Wong, Chi Chiu So, Florence Loong, Lap Ping Chung, William Wai Lung Lam, Raymond Liang, George Kam Hop Li, Dong-Yan Jin, Chor Sang Chim

**Affiliations:** 1 Department of Medicine, Queen Mary Hospital, The University of Hong Kong, Hong Kong; 2 Department of Pathology, Queen Mary Hospital, The University of Hong Kong, Hong Kong; 3 Department of Pathology, Princess Margaret Hospital, Hong Kong; 4 Department of Surgery, Queen Mary Hospital, The University of Hong Kong, Hong Kong; 5 Department of Biochemistry, Queen Mary Hospital, The University of Hong Kong, Hong Kong; Florida International University, United States of America

## Abstract

*miR-124-1* is a tumour suppressor microRNA (miR). Epigenetic deregulation of miRs is implicated in carcinogenesis. Promoter DNA methylation and histone modification of *miR-124-1* was studied in 5 normal marrow controls, 4 lymphoma, 8 multiple myeloma (MM) cell lines, 230 diagnostic primary samples of acute myeloid leukaemia (AML), acute lymphoblastic leukaemia (ALL), chronic myeloid leukaemia (CML), chronic lymphocytic leukaemia (CLL), MM, and non-Hodgkin's lymphoma (NHL), and 53 MM samples at stable disease or relapse. Promoter of *miR-124-1* was unmethylated in normal controls but homozygously methylated in 4 of 4 lymphoma and 4 of 8 myeloma cell lines. Treatment of 5-Aza-2′-deoxycytidine led to *miR-124-1* demethylation and re-expression of mature *miR-124*, which also associated with emergence of euchromatic trimethyl H3K4 and consequent downregulation of CDK6 in myeloma cells harboring homozygous *miR-124-1* methylation. In primary samples at diagnosis, *miR-124-1* methylation was absent in CML but detected in 2% each of MM at diagnosis and relapse/progression, 5% ALL, 15% AML, 14% CLL and 58.1% of NHL (p<0.001). Amongst lymphoid malignancies, *miR-124-1* was preferentially methylated in NHL than MM, CLL or ALL. In primary lymphoma samples, *miR-124-1* was preferentially hypermethylated in B- or NK/T-cell lymphomas and associated with reduced *miR-124* expression. In conclusion, *miR-124-1* was hypermethylated in a tumour-specific manner, with a heterochromatic histone configuration. Hypomethylation led to partial restoration of euchromatic histone code and miR re-expression. Infrequent *miR-124-1* methylation detected in diagnostic and relapse MM samples showed an unimportant role in MM pathogenesis, despite frequent methylation found in cell lines. Amongst haematological cancers, *miR-124-1* was more frequently hypermethylated in NHL, and hence warrants further study.

## Introduction

DNA methylation involves the addition of a methyl group to the number 5 carbon of the cytosine ring in the CpG dinucleotide, by catalyzing the cytosine into methylcytosine through DNA methyltransferase [1], [2]. Cancer cells are characterized by global DNA hypomethylation but gene-specific hypermethylation of promoter-associated CpG islands of tumour suppressor genes (TSGs), resulting in transcriptional repression, and hence serve as an alternative mechanism of gene inactivation. Based on a pathway-specific approach, multiple TSGs across pathways including cell cycle regulation, JAK/STAT signalling, WNT signalling, and DAP kinase-associated intrinsic tumour suppression have been shown to be inactivated by gene hypermethylation in leukaemia, lymphoma and multiple myeloma [3], [4], [5], [6], [7], [8], [9], [10], [11].

MicroRNA (miR) is a single-stranded, non-coding RNA molecule of 22–25 nucleotides, which leads to downregulation of target protein expression [12]. miRs are involved in carcinogenesis. miRs can be either oncogenic (oncomir) when TSGs are targeted, or tumour suppressive (tumour suppressor miRs) when oncogenes are targeted [12], [13]. Little is known about the role of hypermethylation of tumour suppressor miRs in haemic cancers.

Recently, *miR-124-1* has been shown to be hypermethylated in multiple cancers [14], [15]. By luciferase assay, *miR-124-1* has been shown to downregulate CDK6 translation by binding on the 3′ untranslated region (3′ UTR) of the *CDK6* mRNA, and also reduce the retinoblastoma protein phosphorylation, thereby demonstrating the tumour suppressor role of *miR-124-1* [14].

In this study, we aimed to study the role of *miR-124-1* methylation in a wide range of haematological malignancies including acute myeloid leukaemia (AML), chronic myeloid leukaemia (CML), acute lymphoblastic leukaemia (ALL), chronic lymphocytic leukaemia (CLL), multiple myeloma (MM) and non-Hodgkin's lymphoma (NHL).

## Materials and Methods

### Patient samples

Diagnostic bone marrow or tissue samples were obtained in 20 ALL, 20 AML, 11 CML in chronic phase, 50 CLL, 55 MM and 74 NHL patients. Diagnosis of leukaemia and lymphoma were made according to the French-American-British Classification and WHO Classification of Tumours respectively [16], [17], [18], [19]. Of the 20 ALL patients, there were eleven male and nine female patients with a median age of 35 years (range: 13–62). There were six common ALL, one early B precursor, ten precursor B ALL and three pre-T ALL. Of the AML patients, there were nine male and eleven female with a median age of 41.5 years (range: 20–72). The AML cases comprised three M1, fourteen M2, two M4 and one M5 subtype. Of the 50 CLL patients, there were twenty three (46%) patients with limited stage (<stage II) and twenty seven (54%) with advanced stage (≥stage II) disease with a median age of 65.5 years (range: 37–91) [3]. Forty (80%) were male. The median presenting lymphocyte count was 17×10^9^/L (range: 10–236×10^9^/L). Of the 55 MM patients, the median age was 57 (25–87) years. The diagnosis of MM was based on standard criteria [20]. Apart from five patients with insufficient clinical data, there were seven (14%) Durie-Salmon stage I, thirteen (26%) stage II, and thirty (60%) stage III patients. In order to study if *miR-124-1* methylation might be acquired at the time of relapse or after repeated chemotherapy, a total of 53 serial samples from 12 MM patients including those at stable disease, refractory relapse, relapse, primary refractory disease or refractory disease progression were included. Of the 74 patients with NHL, there were 17 patients with peripheral T cell lymphoma (two anaplastic large cell [ALCL], four angio-immunoblastic T-cell [AITL], eleven peripheral T-cell, not otherwise specified [PTCL, NOS]), 10 with natural killer (NK)/T-cell lymphoma, 47 patients with B-cell lymhoma (twenty-one follicular: grade 1 to 2, eight nodal marginal zone, three mantle cell lymphoma and fifthteen diffuse large B-cell lymphoma). The study has been approved by Institutional Review Board of Queen Mary Hospital with informed consent.

### Cell lines and culture

Lymphoma (SU-DHL-1, SUP-M2, SUP-T1, and KARPAS-299) and MM (KMS-12-PE, MOLP-8, OPM-2, and U-266) cell lines were purchased from Deutsche Sammlung von Mikroorganismen und Zellkulturen GmbH (DMSZ) (Braunschweig, Germany). LP-1 and RPMI-8226 were kind gifts from Dr Orlowski (Department of Hematology/Oncology, MD Anderson Cancer Center, USA). WL-2 was kindly provided by Dr Andrew Zannettino (Myeloma and Mesenchymal Research Laboratory, Division of Haematology, Institute of Medical and Veterinary Science, Adelaide, Australia). NCI-H929 was purchased from American Type Culture Collection (ATCC). Cell cultures were maintained in RPMI media 1640 (IMDM for LP-1) (Invitrogen, Carlsbad, CA, USA), supplemented with 10% fetal bovine serum (Invitrogen, Carlsbad, CA, USA), 50 U/ml penicillin, and 50 µg/ml streptomycin (Invitrogen, Carlsbad, CA, USA) in a humidified atmosphere of 5% CO_2_ at 37°C.

### Methylation-specific polymerase chain reaction (MSP)

DNA was extracted from bone marrow samples of ALL, AML, CML, CLL, and MM at diagnosis, diagnostic tissues (either lymph node or nasal biopsy in nasal NK-cell lymphoma) in patients with NHL, and lymphoma and MM cell lines by standard method. MSP for aberrant gene promoter methylation was performed as previously described [8]. Treatment of DNA with bisulfite for conversion of unmethylated cytosine to uracil (but unaffecting methylated cytosine) was performed with a commercially available kit (EpiTect Bisulfite Kit, QIAGEN, Hilden, Germany). Primers used for the methylated MSP (M-MSP) and unmethylated MSP (U-MSP) were published previously [14]. DNA from normal bone marrow donors (N = 5) was used as negative control, while enzymatically methylated control DNA (CpGenome Universal Methylated DNA, Chemicon, Temecula, CA, USA) was used as positive control in all the experiments.

### Bisulfite genomic sequencing (BGS)

Bisulfite-treated DNA was used as template. Promoter region of *miR-124-1* was amplified and cloned using TOPO TA Cloning Kit (Invitrogen, Carlsbad, CA, USA) according to the manufacturer's instructions. Primers used were published previously [14].

### 5-Aza-2′-deoxycytidine (5-AzadC) treatment

For treatment with 5-AzadC (Sigma-Aldrich, St. Louis, MO, USA), cells were seeded in six-well plates at a density of 1×10^6^ cells/ml, and cultured with 1 µM of 5-AzadC for 3 days. Cells on day 0 and day 3 of 5-AzadC treatment were harvested.

### RNA isolation and stem-loop reverse transcription-polymerase chain reaction (RT-PCR)

Total RNA was isolated using *mir*Vana™ miR Isolation Kit (Ambion, Austin, TX, USA), according to the manufacturer's instructions. RT was performed using Taqman® MicroRNA RT Kit and Taqman® MicroRNA Assay Kit (Applied Biosystems, Foster City, CA, USA), according to the manufacturer's instructions. Total RNA was reverse transcribed in 1 mM dNTPs, 50 U MultiScribe™ Reverse Transcriptase, 1× RT Buffer, 3.8 U RNase Inhibitor, and 1× stem-loop RT primer at following thermal cycling condition: 16°C for 30 minutes, 42°C for 30 minutes, and 85°C for 5 minutes. Quantitative real-time PCR of *miR-124* was performed using 1.33 µl of 1∶15 diluted RT product in 1× Taqman® Universal PCR Master Mix, and 1× Taqman® Assay at 95°C for 10 minutes, followed by 40 cycles of 95°C for 15 seconds and 60°C for 1 minute. RNU48 was used as reference for data analysis using the 2^−ΔΔCt^ method [21].

### Chromatin immunoprecipitation (ChIP)

ChIP assays were conducted according to manufacturer's instructions (Upstate, Cat# 17-610). Cells of 2×10^6^ were fixed in 1% formaldehyde for each ChIP. Fixed cells were washed by cold PBS, resuspended in lysis buffer, and sheared into fragments ranging between 200 and 800 bp in size on ice using 431A cup horn (Misonix, Farmingdale, NY, USA). ‘Input’ of 1% was reserved as control, immunoprecipitation was performed by 4°C overnight incubation with anti-H3K4me3 (Upstate, 04-745), anti-H3K9me3 (Upstate, 17-625; Abcam, 8898), anti-H3K9ac (Upstate, 17-658), anti-H3K27me3 (Upstate, 17-622) and normal rabbit IgG respectively. Immunoprecipitated complex was collected by magnetic protein A beads. The complex was washed, treated with proteinase K, and reverse cross-linked by heat. Primers used for ChIP-PCR of *miR-124-1* were forward: 5′- CAA AGA GCC TTT GGA AGA CG -3′ and reverse: 5′- GGA AGA GGG GTG GGT AGA AG -3′. ChIP-PCR was also controlled by *GAPDH* promoter and *Alu* repeats [22], [23].

### Western blot for CDK6

Cells were harvested and lysed in RIPA buffer (50 mM Tris-HCl, pH 7.4, 150 mM NaCl, 0.2% SDS, 1% Triton X-100, 2 mM EDTA) supplemented with protease inhibitors including 4 µg/ml aprotinin, 2 µg/ml leupeptin, 1 µg/ml pepstatin A, 20 µg/ml PMSF, and 1 mM Na_3_VO_4_. Cell debris was removed by centrifugation at 10,000×g for 5 minutes at 4°C. Protein lysate was denatured in an equal volume of loading buffer (100 mM Tris-HCl, pH 6.8, 200 mM DTT, 4% SDS, 0.2% bromophenol blue, 20% glycerol), heated at 95°C for 5 minutes, and separated on 10% SDS-PAGE. Separated samples were then transferred to a 0.2 µm nitrocellulose membrane (Bio-Rad, Hercules, CA). The membrane was blocked at room temperature for 1 hour in 5% skim milk diluted in PBS-Tween 20 (0.5% v/v). The membrane was then incubated with CDK6 primary antibody (1∶1000) at 4°C overnight with shaking. After washing 3 times of 15 minutes each in PBS-Tween 20 (0.5% v/v), the membrane was incubated with anti-mouse horseradish peroxidase conjugate secondary antibody (1∶1000) at room temperature for 1 hour. After washing 3 times of 15 minutes each in PBS-Tween 20 (0.5% v/v), signals were detected by ECL Western blotting detection reagents (Amersham Biosciences, Buckinghamshire, UK) and exposed to X-ray film.

### Statistical analysis

The frequency of *miR-124-1* methylation in different types of haematological cancers was computed by Chi-Square or Fisher Exact test. In CLL, correlation between *miR-124-1* methylation status with continuous (mean age, mean diagnostic haemoglobin, lymphocyte and platelet counts) and categorical variables (gender and Rai staging) were studied by Student *t*-test and Chi-square test (or Fisher Exact test) respectively. Overall survival (OS) is measured from the date of diagnosis to the date of last follow-up or death. OS of patients with limited Rai stage (stages 0, I and II) were compared to those with advanced stage (stage III and IV). Survival is plotted by the Kaplan-Meier method and compared by the log-rank test. All p-values were two-sided. In NHL, correlation between *miR-124-1* methylation with continuous (mean age) and categorical variables (gender, histological subtypes, lineage [B, T or NK/T] and nodal/extranodal presentation) were studied in 49 patients with complete clinical data by Student's *t*-test and Chi-square test (or Fisher Exact test) respectively. Moreover, in 25 primary B-cell NHL samples in which both DNA and RNA were available, the mean expression of *miR-124* in methylated and unmethylated lymphoma were compared by the Student's *t*-test.

## Results

### MSP

#### Controls

**Figure 1:**
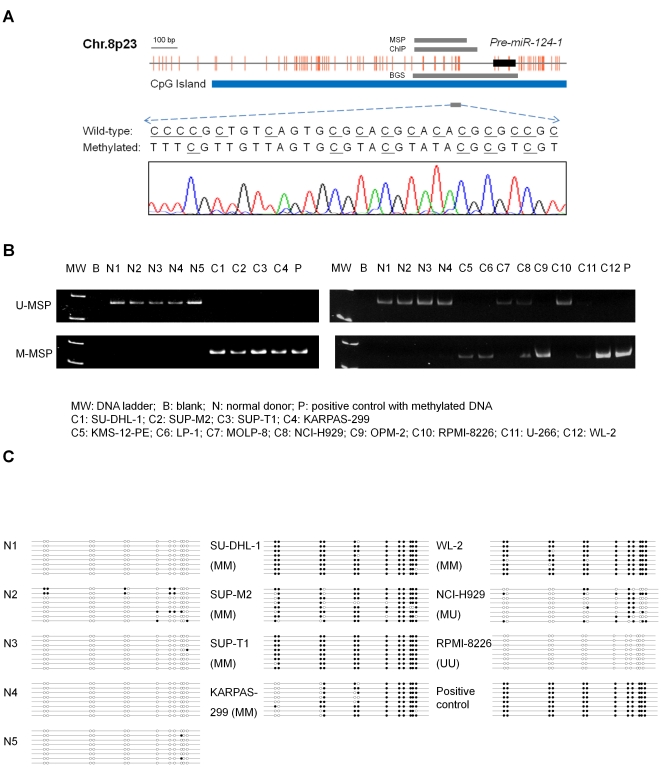
Methylation of *miR-124-1*. (A) Schematic diagram showing the distribution of CpG dinucleotides (solid vertical lines) over the precursor (solid black box) and mature *miR-124-1*. Sequence analysis of the M-MSP product from bisulfite-treated positive control DNA showed that the cytosine [C] residues of CpG dinucleotides were methylated and remained unchanged, whereas all the other C residues were unmethylated and were converted to thymidine [T], indicating complete bisulfite conversion and specificity of MSP. Grey bars indicated the amplification regions of the MSP, ChIP, and BGS primers. (B) U-MSP showed that the methylated positive control [P] was totally methylated, and all five normal controls (N1–N5) were unmethylated. In the M-MSP, the methylated control was positive (methylated) but all normal controls were negative (unmethylated). For the cell lines, SUP-T1, SUP-M2 (ALK+), SU-DHL-1 (ALK+), KARPAS-299 (ALK+), KMS-12-PE, LP-1, OPM-2, and WL-2 were completely methylated of *miR-124-1*. (C) Bisulfite genomic sequencing for the bisulfite-treated promoter region of *miR-124-1* of normal controls (N1–N5), lymphoma and myeloma cell lines of different methylation statuses (MM, UM, or UU), and the methylated positive control were depicted. Unmethylated (empty circle) and methylated (filled circle) CpG dinucleotides were shown by eight independent clones for each sample.

Direct sequencing of the M-MSP products from the methylated positive control showed the expected nucleotide changes after bisulfite treatment, therefore confirming complete bisulfite conversion and specificity of MSP (Figure 1A). None of the five normal control marrows showed aberrant methylation of *miR-124-1* (Figure 1B). The positive and negative controls showed the expected MSP results (normal DNA: U-MSP positive/M-MSP negative; methylated DNA: U-MSP negative/M-MSP positive).

#### Lymphoma cell lines

The profile of methylation of *miR-124-1* of lymphoma cell lines was shown in Figure 1B. SUP-T1, SUP-M2 (ALK+), SU-DHL-1(ALK+) and KARPAS-299 (ALK+) were homozygously methylated for *miR-124-1*.

#### Myeloma cell lines

The profile of methylation of *miR-124-1* of myeloma cell lines was shown in Figure 1B. Apart from MOLP-8 and RPMI-8226, which were completely unmethylated (UU) of *miR-124-1*, KMS-12-PE, LP-1, OPM-2, and WL-2 were homozygously methylated (MM) for *miR-124-1*, whereas NCI-H929 and U-266 were hemizygously methylated (MU) for *miR-124-1*.

Bisulfite genomic sequencing confirmed *miR-124-1* hypomethylation in five normal controls, hypermethylation in the methylated positive control, and the corresponding methylation statuses (MM, MU, and UU) as detected by MSP (Figure 1C).

#### Primary samples at diagnosis

**Figure 2:**
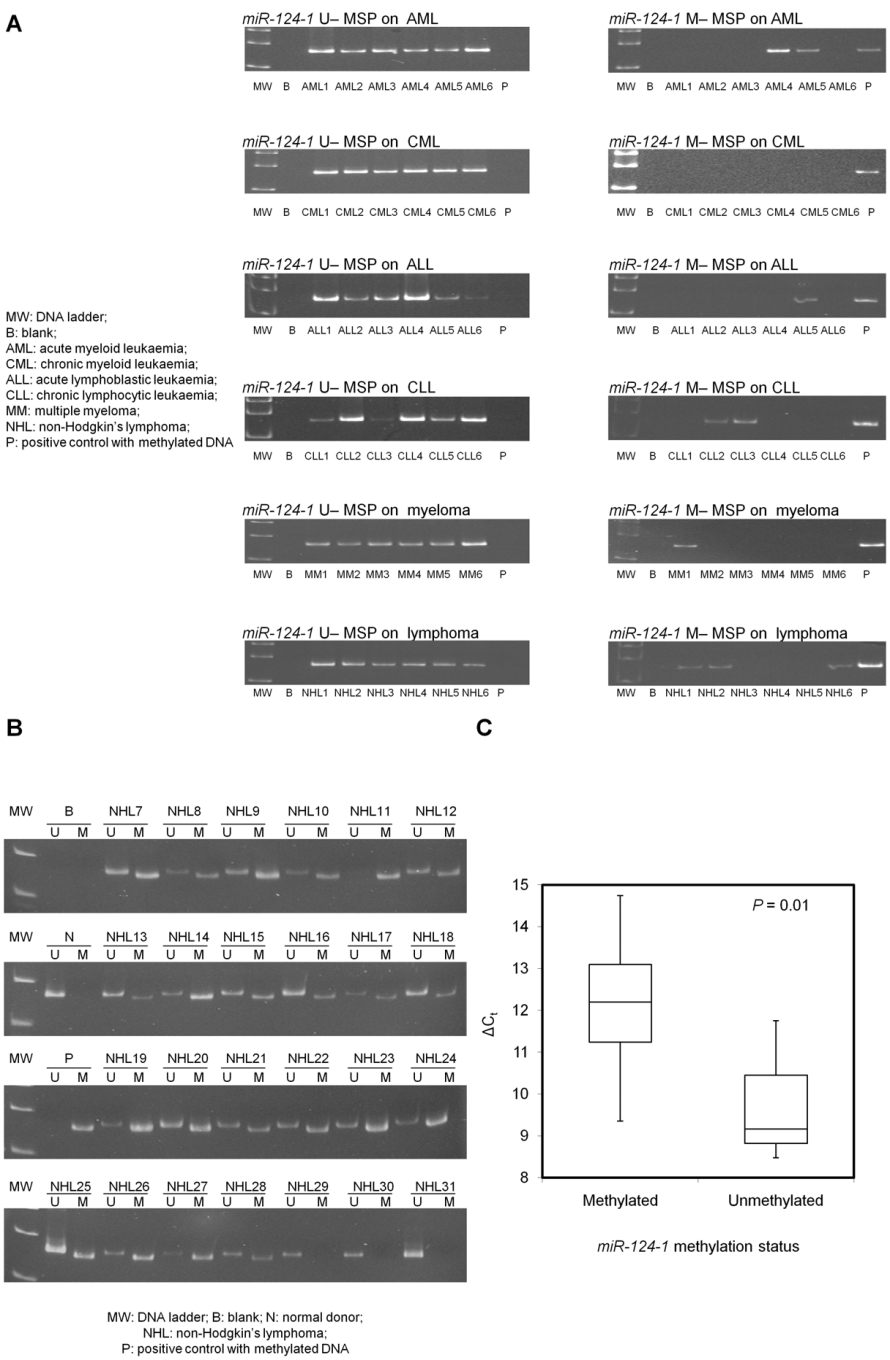
Promoter methylation of *miR-124-1* and expression of *miR-124* in primary samples. (A) Methylation of *miR-124-1* in primary samples. (B) M-/U-MSP analysis of *miR-124-1* promoter methylation status and (C) Stem-loop qRT-PCR analysis of the mature *miR-124* expression in 25 primary NHL samples with matched DNA and RNA. ΔC_t_, C_t_ *miR-124* -C_t_ *RNU48*.

*miR-124-1* hypermethylation was not detected in any of the CML. On the other hand, *miR-124-1* methylation was found in one (2%) MM samples at diagnosis, one (2%) MM samples at relapse/progression, 1 (5%) ALL, 3 (15%) AML, 7 (14%) CLL and 43 (58.1%) NHL samples (p<0.001) (Figure 2A). Amongst the lymphoid malignancies, there was significantly more frequent *miR-124-1* methylation in NHL than MM, CLL or ALL (p<0.001). In CLL, there was no correlation between *miR-124-1* methylation and age (p = 0.79), gender (p = 0.99), diagnostic lymphocyte count (p = 0.89); Hb (p = 0.98), platelet count (p = 0.42), advanced Rai stage (≥stage 2) (p = 0.69) and death (p = 0.41). The projected OS in CLL patients with and without *miR-124-1* methylation were 86% and 62% (p = 0.36). Amongst lymphoma samples, *miR-124-1* was methylated in seven NK/T (70.0%), thirty-one B-cell NHL (66.0%), and five T-cell NHL (29.4%) (p = 0.023). However, *miR-124-1* methylation did not correlate with age (p = 0.457), gender (p = 0.99) or Ann Arbor stage (p = 0.105) of the lymphoma patients.

In order to determine the role of *miR-124-1* methylation on the expression of *miR-124* in primary samples, we analyzed the methylation status and expression level in 25 primary B-cell NHL samples in which both DNA and RNA were available. By MSP and stem-loop qRT-PCR, 22 samples displayed methylated MSP signals and three were completely unmethylated (Figure 2B). Moreover, methylation of *miR-124-1* was associated with a lower level of *miR-124* expression, and hence a higher ΔC_t_ (C_t_ *miR-124* -C_t_ *RNU48*) (p = 0.01) (Figure 2C).

### 5-AzadC treatment of lymphoma and myeloma cells

**Figure 3:**
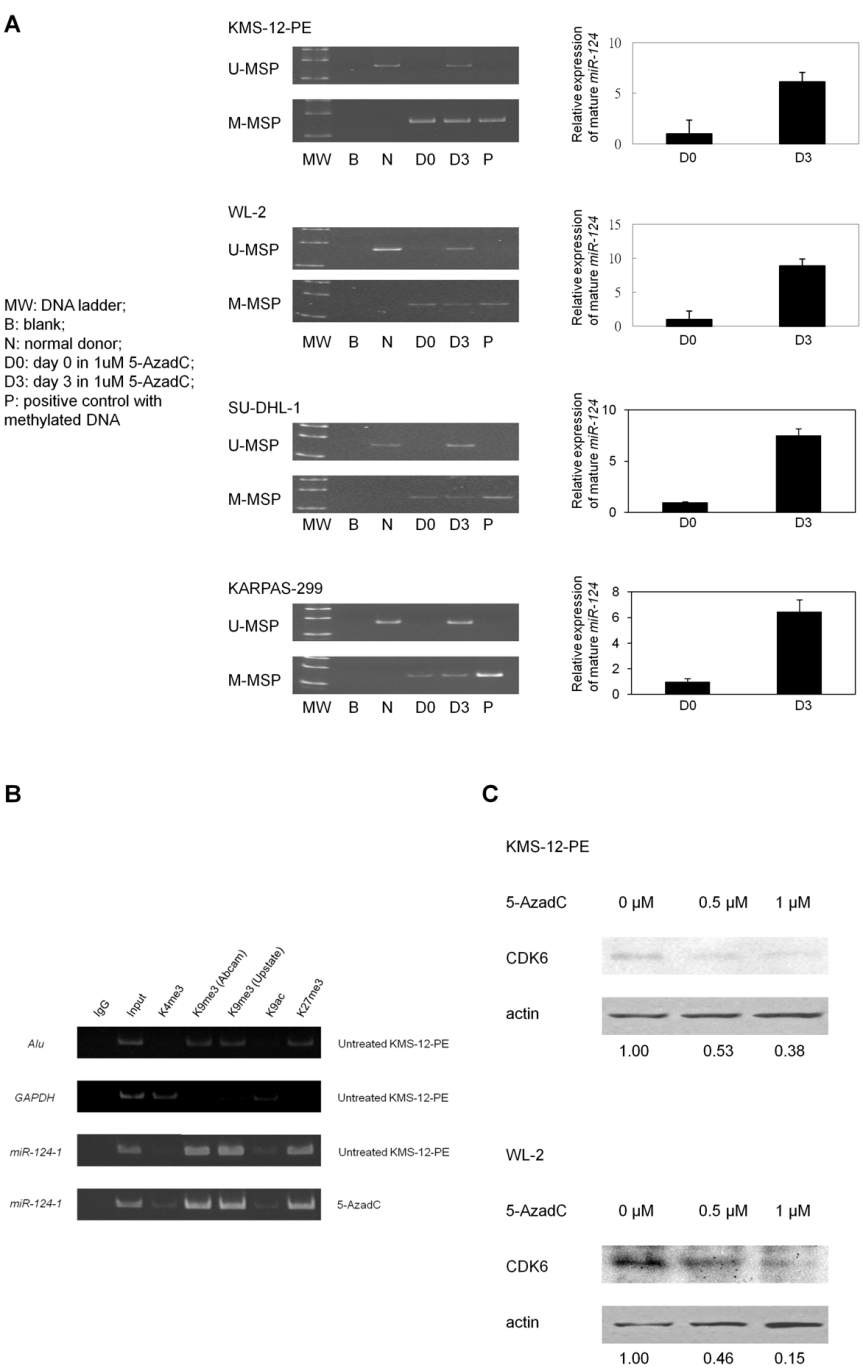
Effect of 5-Aza-2′-deoxycytidine (5-AzadC) treatment on lymphoma and myeloma cells. (A) M-/U-MSP analysis of *miR-124-1* promoter methylation status and stem-loop qRT-PCR analysis of the mature *miR-124* expression. 5-AzadC treatment resulted in progressive demethylation of *miR-124-1* promoter, and re-expression of the mature *miR-124* in cell lines harbouring homozygous *miR-124-1* methylation. (B) ChIP analysis for trimethyl H3K4, trimethyl H3K9, acetyl H3K9, trimethyl H3K27 in *miR-124-1* promoter. 5-AzadC treatment led to augmentation of euchromatin code of trimethyl H3K4. (C) Western blot analysis of CDK6 in response to 5-AzadC treatment. Bottom row showed densitometric quantization of the Western blot, indicating relative CDK6 expression under actin normalization.

SU-DHL-1, KARPAS-299, KMS-12-PE, and WL-2 cells were completely methylated for *miR-124-1*. Upon 5-AzadC demethylation treatment, *miR-124-1* U-MSP signal emerged on day 3, with re-expression of mature *miR-124* as shown by Taqman stem-loop qRT-PCR (Figure 3A). 5-AzadC treatment led to augmentation of euchromatic histone code with abundance of trimethyl H3K4 at *miR-124-1* promoter region (Figure 3B). *GAPDH* promoter and *Alu* repeat element, with the inherent hypo- and hypermethylated DNA, were used as biological controls for euchromatin and heterochromatin configurations (Figure 3B). Finally, demethylation of *miR-124-1* by 5-AzadC with *miR-124* re-expression led to downregulation of CDK6 (Figure 3C).

## Discussion

There are several observations.

Firstly, in this study, we showed that *miR-124-1* is not methylated in normal blood cells but is hypermethylated in lymphoma and myeloma cell lines, which can be re-expressed upon hypomethylating treatment. In cancer, miRs may be hypermethylated by two patterns. First, tumour suppresssor miRs are expected to be hypomethylated in normal cells but hypermethylated in cancer cells [24]. On the other hand, some miRs may be hypermethylated in both normal and tumour cells, and therefore, hypermethylation of these miRs is tissue-specific but not tumour-specific. For example, *miR-127* and *miR-373* are hypermethylated in both the normal and cancer cells [24], [25]. Therefore, our data and those from Lujambio *et al.* showed that *miR-124-1* is differentially methylated in cancer cells but not normal cells, consistent with its tumour suppressor role [14]. In addition to miR silencing associated with *miR-124-1* methylation, miR expression could be restored by *miR-124-1* demethylation, which was associated with restoration of the euchromatin code trimethyl H3K4. Moreover, *miR-124-1* re-expression after hypomethylating treatment was associated with downregulation of CDK6 expression, consistent with data that CDK6 is a target of translation repression by *miR-124-1* [14]. Furthermore, to ensure ChIP specificity, we showed two trimethyl H3K9 antibodies of different preparations generated comparable results, which further controlled the ChIP technically together with input and IgG controls; in addition to *GAPDH* and *Alu* repeat element, with the inherent hypo- and hypermethylated DNA, which served as biological controls for euchromatin and heterochromatin configurations.

Secondly, as *miR-124-1* is localized to chromosome 8p, where loss of heterozygosity (LOH) is frequently found in various solid cancers [26], [27], [28], [29], and certain subtypes of NHL including mantle cell [30], and small B cell lymphoma [31]. Therefore, *miR-124-1* hypermethylation may collaborate with LOH to result in biallelic *miR-124-1* inactivation in NHL, thereby fulfilling the Knudson's hypothesis [32]. This was supported by the finding that *miR-124-1* was preferentially methylated in lymphoma, in particular, in B- and NK/T-cell lymphomas, which was associated with a lower expression of *miR-124*. Therefore methylation of *miR-124-1* might be important in lymphomagenesis.

Fourthly, *miR-124-1* was preferentially hypermethylated in NK/T-cell lymphoma, which is an Epstein-Barr virus–associated, aggressive extranodal lymphoma more frequently encountered in Asia, and Central and South America [33]. Various tumour suppressor genes have been shown to be frequently hypermethylated in NK/T-cell lymphoma including *p73*, *CDKN2A*, *CDKN2B*, *hMLH1* and *RARβ* [34], but hypermethylation of *miR-124-1* is one of the first reports of methylation of miR in NK/T-cell lymphoma. On the other hand, in contrast to frequent (del)8p in B-cell lymphoma, del(8p) is infrequent in NK/T-cell lymphoma [35], and hence, in NK/T-cell lymphomas, *miR-124-1* might be inactivated by biallelic hypermethylation instead of deletion together with gene hypermethylation. Apart from NHL, *miR-124-1* methylation is infrequent in other haematological malignancies in contrast to solid cancer such as colon and lung cancers, in which *miR-124* methylation was detected in 48%–75% of primary samples [14]. On the other hand, in contrast to a previous report of frequent hypermethylation of *miR-124* in ALL [36], only 5% of ALL patients carried hypermethylation of *miR-124-1* in this series. Possible reasons included the small sample size here and the inclusion of merely adult but not paediatric patients in our study.

Finally, despite frequent hypermethylation of *miR-124-1* in myeloma cell lines, *miR-124-1* methylation was found infrequent in diagnostic marrow samples, and hence we postulated that *miR-124-1* methylation may be acquired during disease progression or after repeated chemotherapy. In particular, we included samples after repeated chemotherapy regimens after clinical relapse. However, no significant methylation of *miR-124-1* was demonstrated in relapsed myeloma marrow samples either, even after repeated intensive chemotherapy regimens. Therefore, *miR-124a* methylation is unimportant in the pathogenesis or progression of MM.

In summary, *miR-124-1* hypermethylation is tumour-specific, associated with gene silencing, which can be reversed by hypomethylating treatment. Re-expression of *miR-124* by 5-AzadC treatment was associated with emergence of a partial euchromatin histone code and consequent downregulation of CDK6. Amongst haematological malignancies, *miR-124-1* is preferentially hypermethylated in NHL (in particular NK/T-cell lymphoma), in which methylation of *miR-124-1* was associated with a lower expression of *miR-124*, and hence warrant further study in lymphoma. Finally, in MM, despite frequent *miR-124-1* methylation in myeloma cell lines, *miR-124-1* methylation was infrequent in primary samples including relapse samples, and hence unimportant in myeloma pathogenesis.
